# Diagnostic Ability of Peguero-Lo Presti Electrocardiographic Left Ventricular Hypertrophy Criterion in Severe Aortic Stenosis

**DOI:** 10.3390/jcm10132864

**Published:** 2021-06-28

**Authors:** Aleksandra Gamrat, Katarzyna Trojanowicz, Michał A. Surdacki, Aleksandra Budkiewicz, Adrianna Wąsińska, Ewa Wieczorek-Surdacka, Andrzej Surdacki, Bernadeta Chyrchel

**Affiliations:** 1 Students’ Scientific Group at the Second Department of Cardiology, Jagiellonian University Medical College, 2 Jakubowskiego Street, 30-688 Cracow, Poland; aleksandra.gamrat@gmail.com (A.G.); katarzyna.trojanowicz@student.uj.edu.pl (K.T.); msurdacki1997@gmail.com (M.A.S.); aleksandra.budkiewicz@student.uj.edu.pl (A.B.); ada231.wasinska@gmail.com (A.W.); 2 Chair and Department of Nephrology, Faculty of Medicine, Jagiellonian University Medical College, 2 Jakubowskiego Street, 30-688 Cracow, Poland; esurdacka@gmail.com; 3 Second Department of Cardiology, Institute of Cardiology, Faculty of Medicine, Jagiellonian University Medical College, 2 Jakubowskiego Street, 30-688 Cracow, Poland; surdacki.andreas@gmx.net

**Keywords:** left ventricular hypertrophy, electrocardiography, aortic stenosis

## Abstract

Traditional electrocardiographic (ECG) criteria for left ventricular hypertrophy (LVH), introduced in the pre-echocardiographic era of diagnosis, have a relatively low sensitivity (usually not exceeding 25–40%) in detecting LVH. A novel Peguero-Lo Presti ECG-LVH criterion was recently shown to exhibit a higher sensitivity than the traditional ECG-LVH criteria in hypertension. Our aim was to test the diagnostic ability of the novel Peguero-Lo Presti ECG-LVH criterion in severe aortic stenosis. We retrospectively analyzed 12-lead ECG tracings and echocardiographic records from the index hospitalization of 50 patients with isolated severe aortic stenosis (mean age: 77 ± 10 years; 30 women and 20 men). Exclusion criteria included QRS > 120 ms, bundle branch blocks or left anterior fascicular block, a history of myocardial infarction, more than mild aortic or mitral regurgitation, and significant LV dysfunction by echocardiography. We compared the agreement of the novel Peguero-Lo Presti criterion and traditional ECG-LVH criteria with echocardiographic LVH (LV mass index > 95 g/m^2^ in women and >115 g/m^2^ in men). Echocardiographic LVH was found in 32 out of 50 study patients. The sensitivity of the Peguero-Lo Presti criterion in detecting LVH was improved (55% vs. 9–34%) at lower specificity (72% vs. 78–100%) in comparison to 8 single traditional ECG-LVH criteria. Additionally, the positive predictive value (77% vs. 72%), positive likelihood ratio (2.0 vs. 1.5), and odds ratio (3.2 vs. 2.4) were higher for the Peguero-Lo Presti criterion versus the presence of any of these 8 traditional ECG-LVH criteria. Cohen’s Kappa, a measure of concordance between ECG and echocardiography with regard to LVH, was 0.24 for the Peguero-Lo Presti criterion, −0.01–0.13 for single traditional criteria, and 0.20 for any traditional criterion. However, by the receiver operating characteristics (ROC) curve analysis, the overall ability to discriminate between patients with and without LVH was insignificantly lower for the Peguero-Lo Presti versus Cornell voltage as a continuous variable (area under the ROC curve: 0.65 (95% CI, 0.48–0.81) vs. 0.71 (0.55–0.86), *p* = 0.5). In conclusion, our preliminary results suggest a slightly better, albeit still low, agreement of the novel Peguero-Lo Presti ECG criterion compared to the traditional ECG-LVH criteria with echocardiographic LVH in severe aortic stenosis.

## 1. Introduction

Left ventricular hypertrophy (LVH), defined as increased left ventricular mass, has long been recognized as a predictor of adverse clinical events, including death from cardiovascular causes [1,2,3,4]. In the pre-echocardiographic era, traditional ECG criteria were the only practical technique in the diagnosis of LVH [5]. However, their drawback is a low sensitivity, generally not exceeding 30–40%, in detecting LVH diagnosed by magnetic resonance or echocardiography, the current standards in LVH diagnosis [5,6], Nevertheless, ECG is still used as a screening tool owing to its wide availability. Moreover, recent findings suggest that LVH on ECG and anatomic LVH have independent prognostic values for cardiovascular mortality [6,7,8,9,10].

A novel ECG criterion for LVH, proposed by Peguero et al. [11], was recently shown to be more sensitive than traditional ECG-LVH criteria in patients with arterial hypertension. To date, the ability of the novel criterion to discriminate patients with and without anatomic LVH have been estimated in various clinical settings, including cardiac patients and the general population, demonstrating generally rather modest (and, at best, moderate) superiority over the classical ECG-LVH criteria in some [12,13,14,15,16], but not all [17,18,19,20,21,22,23]. However, to the best of our knowledge, only one study was focused on patients with aortic stenosis [24], the most prevalent valvular heart disease. Nevertheless, although in that report the Peguero-Lo Presti criterion had higher sensitivity than Cornell and Sokolow–Lyon LVH voltage criteria to predict anatomic LVH, a complex analysis of the agreement between the novel criterion, traditional ECG-LVH criteria, and anatomic LVH in aortic stenosis has not been reported so far.

Our aim was to compare the concordance of the novel Peguero-Lo Presti LVH criterion and traditional ECG-LVH criteria with echocardiographic LVH in severe aortic stenosis.

## 2. Materials and Methods

Out of 83 pre-screened subjects previously hospitalized in our center with the final diagnosis of severe aortic stenosis by means of echocardiography [25], we selected 50 previously described subjects (30 women and 20 men; average age 77 ± 10 years) with isolated severe aortic stenosis without relevant coexistent diseases [26]. As reported [26], we excluded subjects with QRS duration >120 ms, His bundle branch or left anterior fascicular block, more than mild aortic regurgitation or disease of another valve, a history of myocardial infarction, and left ventricular (LV) ejection fraction below 40%.

We compared the agreement of the novel Peguero-Lo Presti criterion [11] and traditional ECG-LVH criteria on routine in-hospital 12-lead ECG tracing [5,26] with echocardiographic LVH, defined in accordance with current recommendations as a LV mass (by the Devereux equation) over 95 g/m^2^ in women and over 115 g/m^2^ in men [27].

Traditional ECG-LVH criteria include R wave amplitudes (R I > 1.5 mV, R aVL ≥ 1.1 mV, max. R V5/V6 > 2.6 mV), R V6 > R V5 (Holt–Spodick criterion), and the combined voltage criteria introduced by Sokolow and Lyon (S V1 + max. R V5/V6 > 3.5 mV), Romhilt (S V2 + max. R V5/V6 > 4.5 mV), Cornell (R aVL + S V3 > 2.0 in women or >2.8 mV in men) as well as Gubner and Ungerleider (R I + S III > 2.5 mV) [5]. We also calculated the novel Peguero-Lo Presti voltage criterion, defined as the sum of the amplitude of the deepest S wave in any lead and the S wave in lead V4 ≥ 2.8 mV in men and ≥2.3 mV in women [11].

The protocol was approved by the institutional ethics committee, including the fact that patients’ informed consent was not obtained due to a retrospective design (Approval No.: 1072.6120.260.2020 issued on 24 September 2020).

Beyond sensitivity, specificity, positive predictive value (PPV), and negative predictive value (NPV), we calculated positive likelihood ratio, odds ratio, and relative risk as estimates of the diagnostic ability of ECG-LVH criteria with regard to echocardiographic LVH. Positive likelihood ratio was defined as the probability of having LVH on ECG in patients with versus without echocardiographic LVH, i.e., sensitivity/(1 − specificity), odds ratio as the odds of echocardiographic LVH (i.e., the probability of having/not having echocardiographic LVH) in patients with versus without LVH on ECG, while relative risk as the probability of echocardiographic LVH in patients with versus without LVH on ECG.

Additionally, we computed Cohen’s kappa to estimate concordance of echocardiographic LVH and LVH on ECG, or mutual agreement between two different ECG-LVH criteria, corrected for the agreement which might be expected by random chance [28]. The presence of a systematic difference between echocardiographic LVH and the ECG-LVH criteria was assessed by McNemar’s test. A Cohen’s kappa of 0.01–0.20, 0.21–0.40, 0.41–0.60, 0.61–0.80, and >0.8 is equivalent to slight, fair, moderate, substantial, and almost perfect, respectively, degree of concordance between the results obtained by two different methods [28].

Additionally, since by means of the receiver operating characteristics (ROC) curve analysis we previously observed the highest ability of the Cornell voltage to predict echocardiographic LVH among 13 classical LVH-related QRS voltages in our study group [26], the diagnostic performance of the Peguero-Lo Presti voltage against the Cornell voltage was also compared as a continuous variable by the ROC curve analysis.

Finally, using multiple regression, we estimated potential confounding effects of transaortic pressure gradient, averaged in-hospital mean arterial blood pressure, age, gender, and body mass index on the association between LV mass index and the Peguero-Lo Presti voltage.

All statistical analyses were made with STATISTICA version 13.3.704.0 (TIBCO Software Inc., 2017; Palo Alto, CA, USA).

## 3. Results

Patients’ characteristics are presented in Table 1.

The sensitivity of the Peguero-Lo Presti criterion in predicting echocardiographic LVH (present in 32 of 50 study subjects, out of whom 90% presented concentric LVH [24]) was improved (55%) in comparison to 8 single traditional ECG-LVH criteria (9–34%) at lower specificity (72% vs. 78–100%) (Table 2). In addition, NPV (48% vs. 36–41%) and overall accuracy (61% vs. 38–50%) were slightly higher for the Peguero-Lo Presti criterion than the 8 classical ECG-LVH criteria analyzed separately (Table 2).

Additionally, PPV (77% vs. 72%), positive likelihood ratio (2.0 vs. 1.5) and odds ratio (3.2 vs. 2.4) were higher for the Peguero-Lo Presti compared to the presence of any of these 8 traditional criteria (Table 2 and Table 3).

Regarding the presence of echocardiographic LVH, Cohen’s kappa was 0.24 for the Peguero-Lo Presti criterion, −0.01–0.13 for single traditional ECG criteria analyzed separately, and 0.20 for the presence of any of the traditional criteria (Table 2).

**Figure 1:**
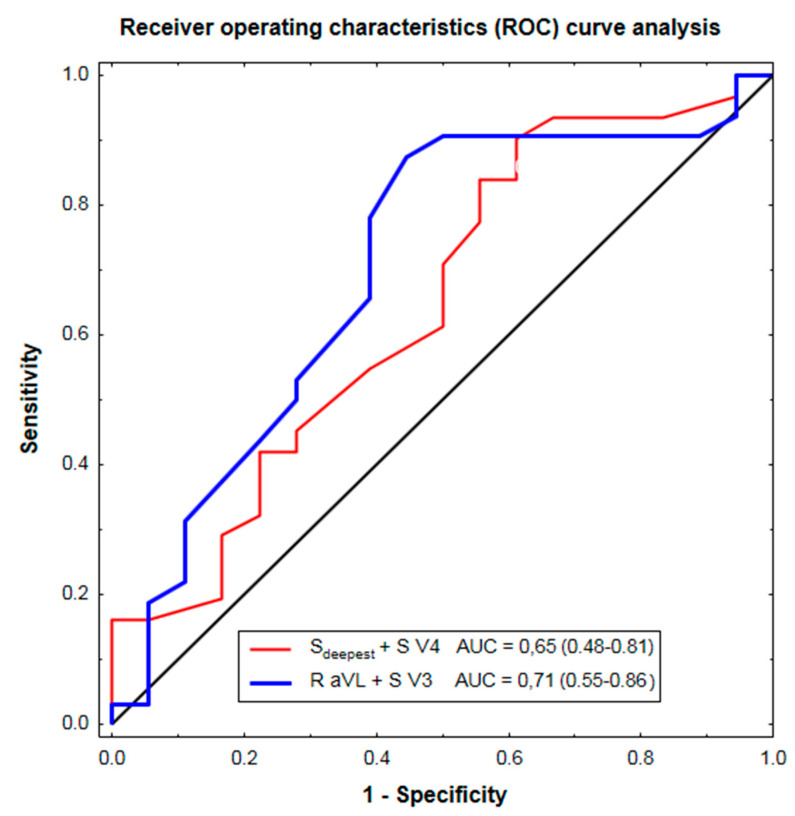
The ability of the Peguero-Lo Presti (S_deepest_ + S V4) and Cornell (R aVL + S V3) voltage as a continuous variable to discriminate aortic stenosis patients with and without echocardiographic LVH, depicted as mean area under the ROC curve (AUC) and 95% confidence interval.

However, mean area under ROC curve (AUC [95% confidence interval]) was insignificantly lower for the Peguero-Lo Presti compared to the Cornell voltage (0.65 (0.48–0.81) vs. 0.71 (0.55–0.86), *p* = 0.5) (Figure 1), whose AUC was highest among the traditional voltages as previously shown [26].

The degree of agreement between the Peguero-Lo Presti criterion and the traditional ECG-LVH criteria was highest for the Cornell voltage, followed by the Sokolow–Lyon voltage, Romhilt voltage, R aVL, Holt-Spodick criterion, Gubner–Ungerleider voltage, max. R V5/V6 and R I (Cohen’s kappa: 0.28, 0.18, 0.16, 0.12, 0.07, 0.0, −0.02, and −0.05, respectively).

By multiple regression (adjusted coefficient of multiple determination (*R^2^*), 0.30, *p* < 0.001), the Peguero-Lo Presti voltage was associated with gender (men vs. women: mean non-standardized regression coefficient, 0.70 ± 0.27, *p* = 0.01) and LV mass index (mean standardized regression coefficient (β) ± standard error of the mean, 0.30 ± 0.13, *p* = 0.03), being unrelated to peak aortic pressure gradient (β = 0.17 ± 0.13, *p* = 0.2), averaged in-hospital mean arterial pressure (β = 0.15 ± 0.13, *p* = 0.3), age (β = −0.23 ± 0.12, *p* = 0.06), and body mass index (β = −0.03 ± 0.13, *p* = 0.8).

## 4. Discussion

Our results suggest a slightly better—albeit still rather low—ability of the Peguero-Lo Presti criterion to predict echocardiographic LVH than the traditional ECG-LVH criteria in severe isolated aortic stenosis.

A higher sensitivity of the Peguero-Lo Presti criterion compared to the traditional criteria in predicting LVH (62% vs. 35%) was reported for the first time in 2017 among patients with arterial hypertension at a similar specificity (~90%) [11]. It was postulated that a greater accuracy of the novel criterion might result, inter alia, from the measurement of the deepest S waves in any ECG lead, not in fixed leads [11,23]. This flexibility is likely to attenuate the interference of such variables as body habitus, distance between the heart and the skin, and tissue electrical conductivity, all of which can non-uniformly influence the voltage recorded by ECG electrodes in different leads. Moreover, Peguero et al. [11] suggested that the second part of the QRS complex, corresponding to the S wave, may have a better association with LV mass, because it reflects the propagation of the depolarization wave front through intramyocardial and epicardial fibers of the LV free wall, which changes the mean depolarization vector already in patients with mild LVH in the horizontal plane, represented by precordial ECG leads. Additionally, in the same study group as the present study, we recently demonstrated a higher predictive value of S waves versus R waves with regard to echocardiographic LVH in both precordial and limb leads by means of the ROC curve analysis across all possible threshold voltage values as continuous predictors [26]. Accordingly, as R waves reflect earlier phases of ventricular depolarization than S waves, their net amplitude is more influenced by multiple competing vectors which can partially cancel each other, thereby decreasing R wave voltage and its association with LV mass.

In our hands, the Peguero-Lo Presti criterion exhibited the highest sensitivity, followed by the Cornell criterion (55% vs. 34% for the Peguero-Lo Presti and Cornell criterion, respectively), in agreement with the pivotal retrospective study by Peguero et al. [11], a report by Ramchand et al. [24] in aortic stenosis (48–49% vs. 24–26%) and prospective studies by Keskin et al. [23] in 310 hypertensive subjects (19% vs. 12%) and Guerreiro et al. [13] in 240 consecutive cardiac patients referred for cardiac magnetic resonance (47% vs. 29%). However, in the present study this benefit was achieved at the cost of reduced specificity, in contrast to preserved specificity in those studies (90% vs. 92% [11], 84–92% vs. 82–92% [24], 93% vs. 94% [23] and >94% [13]). This discrepancy is unlikely a consequence of different patients’ demographics because the specificity of the novel criterion is high in older patients [14,29], as our study subjects. An improved sensitivity of the Peguero-Lo Presti versus Cornell criterion (42–57% vs. 19–21%) at reduced specificity (67–83% vs. 96%) was also observed by Sun et al. [19] in a large Asian general population, out of whom about 50% had hypertension, which was a likely cause of a slightly lower discriminating ability of the Peguero-Lo Presti voltage by the ROC curve analysis in that report (0.665 vs. 0.699 in men and 0.689 vs. 0.721 in women) [19] and in the present study.

These inconsistencies could result from confounding effects of some interfering factors, such as the LV remodeling pattern, stenosis severity, or LV dysfunction which might attenuate the relationship between LVH on ECG and echocardiography. First, concentric LVH was linked to improved sensitivity of some ECG-LVH criteria [30,31]. Nevertheless, concentric LVH, predominant in our patients and in the study by Peguero et al. [11], accounted for only 61% of LVH in the report by Keskin et al. [23]. On the other hand, Ye et al. [32] observed more pronounced associations of the Peguero-Lo Presti than Cornell voltage with relative LV wall thickness beyond LV mass. This could preferentially impair the specificity of the Peguero-Lo Presti criterion via false positive LVH diagnosis in patients with concentric LV geometry, common in aortic stenosis.

Second, peak transvalvular velocity or pressure gradient were previously shown to influence ECG voltage criteria irrespective of LV mass [26,33,34]. Importantly, both Bula et al. [34] and our study group [26] have recently described positive associations of the Sokolow–Lyon and Romhilt voltage but not Cornell voltage with peak aortic jet velocity [34] or gradient [26] by multiple regression. Accordingly, the lack of such correlations for the Peguero-Lo Presti voltage in the same study group might have enhanced the diagnostic performance of the Peguero-Lo Presti voltage against the voltages other than the Cornell voltage. That we observed lower AUC for the Peguero-Lo Presti voltage compared to the Cornell voltage is consistent with the proposed hypothesis.

Third, a low final number of patients analyzed in the present study strongly limits the interpretation of our results. However, in order to limit potential confounding effects of coexistent diseases we had excluded subjects with a history of myocardial infarction, relevant heart valve disorders other than aortic stenosis or LV ejection fraction below 40%. Nevertheless, subclinical LV dysfunction, frequently accompanying aortic stenosis despite preserved global LV systolic function [35], was shown to increase the Sokolow–Lyon and Cornell voltages regardless of LV mass in hypertensive subjects [36]. Moreover, relations between the Peguero-Lo Presti voltage and the degree of subclinical LV dysfunction have not been described so far, to the best of our knowledge. However, owing to a retrospective study design based on routine medical records, we were not able to perform such an analysis because novel echocardiographic techniques were unavailable for the vast majority of the study patients.

## 5. Conclusions

Our preliminary results suggest a slightly better, albeit still low, agreement of the novel Peguero-Lo Presti ECG criterion than the traditional ECG-LVH criteria with echocardiographic LVH in severe aortic stenosis. Larger studies are warranted to validate the Peguero-Lo Presti LVH criterion and identify its potential confounders in patients with LVH of different etiologies.

## Figures and Tables

**Table 1:** Patients’ characteristics.

Characteristic	Mean ± SD or n (%)
Age, years	77 ± 10
Women/men, n	30/20
Hypertension, n (%)	46 (92%)
Mean arterial pressure, mm Hg	94 ± 11
Diabetes, n (%)	26 (52%)
Body mass index, kg/m^2^	26.9 ± 4.2
eGFR, mL/min/1.73 m^2^	70 ± 16
LV mass index, g/m^2^	121 ± 39
LV end-diastolic diameter, mm	46 ± 7
Relative LV wall thickness	0.54 ± 0.13
Aortic valve area, cm^2^	0.7 ± 0.2
Mean aortic gradient, mm Hg	53 ± 19
Peak aortic gradient, mm Hg	85 ± 27

Abbreviations: eGFR: estimated glomerular filtration rate by the CKD-EPI formula; LV: left ventricular; n: number; SD: standard deviation.

**Table 2:** Diagnostic performance of the Peguero-Lo Presti criterion and traditional ECG-LVH criteria to predict echocardiographic LVH.

ECG Criteria for LVH	Sensitivity	Specificity	PPV	NPV	Accuracy	Cohen’s Kappa	McNemar Test
Traditional ECG-LVH criteria							
Single traditional criteria	9–34%	78–100%	60–100%	36–41%	38–50%	−0.01–0.13	≤0.0014
≥1 traditional criterion	66%	56%	72%	48%	62%	0.20	0.6
Peguero-Lo Presti criterion							
S_deepest_ + S V_4_ ≥ 2.3 mV (W)							
S_deepest_ + S V_4_ ≥ 2.8 mV (M)	55%	72%	77%	48%	61%	0.24	0.07

LVH: left ventricular hypertrophy; PPV: positive predictive value; NPV: negative predictive value; W: women; M: men.

**Table 3:** Positive likelihood ratio of electrocardiographic LVH in patients with versus without echocardiographic LVH, and odds ratio and relative risk of echocardiographic LVH in patients with versus without any of the traditional ECG-LVH criteria.

LVH Criteria by ECG	Positive Likelihood Ratio	Odds Ratio (95% CI)	Relative Risk (95% CI)
**≥1 traditional criterion**	1.5	2.4 (0.7–7.8)	1.4 (0.6–3.5)
**Peguero-Lo Presti criterion**			
S_deepest_ + S V_4_ ≥ 2.3 mV (W) S_deepest_ + S V_4_ ≥ 2.8 mV (M)	2.0	3.2 (0.9–11.0)	1.5 (0.6–3.7)

CI: confidence interval; other abbreviations as in Table 1.

## Data Availability

The data presented in this study are available on request from the corresponding author.
